# Beta-Eucaine for Local Anesthesia

**Published:** 1904-03

**Authors:** 


### Beta-Eucaine for Local Anesthesia.

F. Gregory Cornell, M. D., Surgeon to St. Vincent's Hospital, Leadville, Colo., (*Annals of Surgery,* December, 1903,) says that local anesthesia is usually accomplished by freezing or by the injection of cocaine or analogous preparations. The former is most unsatisfactory, owing to the pain of the freezing and thawing and the tissue changes which it causes. The chief reason for the existence of the cocaine substitutes is the danger with cocaine of systemic poisoning from its absorption. Out of 250 reported cases of cocaine poisoning, thirteen terminated fatally. The author himself had three cases of evil effects in a series of fifty cocaine injections, while in a series of over eighty cases in which beta-eucaine was the anesthetic used, no such symptoms were manifest.

Beta-eucaine has for its most characteristic and advantageous features the following: (*a*) nontoxicity, the fatal does being between 6 and 7½ grains per 2 1-5 pounds of body weight, there is practically no possibility of such a dose being injected in the course of an ordinary anesthesia; (*b*) it may be sterilised by heat without the loss of any of its properties; (*c*) it will not deteriorate or decompose with keeping; and (*d*), it will not increase the tendency to hemorrhage to any marked degree; vasomotor paralysis and secondary hemorrhage occur less frequently than with cocaine. These points have been most influential in placing beta-eucaine as the local anesthetic of choice, and its use is being rapidly increased.

The author further advocates infiltration anesthesia according to Schleich and that according to Braun, the latter combining adrenalin with cocaine or eucaine for this purpose. This combination has the advantage of a hemostatic action and of intensifying the anesthesia. Regional anesthesia is also highly recommended. It is the injection of 1 to 3 per cent. anesthetic solution into the sensory nerve that supplies the field of operation, usually at a convenient point between the central nervous system and the site of operative interference, though in some cases the nerves may be injected when exposed in the operative wound. A combination of infiltration and regional methods is usually employed, the former for the skin and the superficial parts and the latter by the direct injection into the nerve. When it is inconvenient to expose the nerve by dissection, an injection into the perineural tissue will, as a rule, be found ample and sufficient. The injection should be made into the region of the nerve to be interfered with and the nerve surrounded by what Matas terms an "anesthetic atmosphere." This "blocking," as shown by the splendid work of Crile and of Cushing on the subject of shock, also prevents centripetal impulses from reaching the center, thus removing one of the most important, if not the chief, causes of shock.

The advantages of local over general anesthesia are readily apparent. The former should be used as an adjuvant to, when not possible as a supplanter of, the latter. As von Mikulicz has said, "the question of today is not which is the safer anesthetic, chloroform or ether, but in what cases can local anesthesia be substituted for anesthesia by inhalation."

